# Anæsthesia in American Hospitals

**Published:** 1912-09-21

**Authors:** 


### ANÆSTHESIA IN AMERICAN HOSPITALS.

It will not be denied by anyone who has followed the developments of the last few years in the science and art of anæsthesia that in the United States of America there are anæsthetists of the highest skill, whose discoveries and labours reflect great credit both upon themselves and upon the American medical profession. It suffices to mention the name of Dr. R. H. Ferguson to prove this point; and we mention him because his work is, perhaps, better known in this country than that of any other American anæsthetist, not because his eminence in this specialty places him beyond comparison with others who could be named.

Yet notwithstanding this indisputable fact, that America produces first-class anæsthetists, whose researches have markedly advanced the subject; notwithstanding his, it is true that the average level of proficiency in the administration of anæsthetics attained there is deplorably low, certainly very much lower than is the case in this country. In making this assertion one feels quite secure from criticism, because the fact is admitted by many prominent surgeons and anæsthetists in the United States. The fact is deplored, no attempt is made to conceal it, and measures are discussed for bringing about an alteration. This state of feeling is hopeful; it shows that a weak spot has been recognised, and that there is no intention of leaving it unremedied. It is pleasant to feel that in Britain we do things better in this connection; and it is sincerely to be hoped that we shall evince as earnest an intention to improve our own shortcomings, both in this and in other portions of medical training and education.

One very pressing reform is the substitution of qualified for unqualified anæsthetists in American hospitals. Even in large hospitals it is not uncommon for a nurse to act as routine anæsthetist; and even when things are not quite so bad as this, the employment of regular anæsthetists is quite exceptional. Any recently qualified resident medical officer or even an undergraduate student is regarded as capable of taking this part in the operation. Now it is true that many young residents give anæsthetics, both in this country and in America, and do it very well. But the universal employment of trained anæsthetists in our teaching hospitals, and indeed in all our large voluntary hospitals, results in a much more thorough teaching and training for the students; and this means that our junior resident medical officers know a good deal about the first principles of anæsthesia. Not only so, some modicum of practical experience (under expert supervision) is now required of the medical student before he presents himself for his professional qualifying examination. It is satisfactory to know that some progress is being made with respect to the conditions under which anæsthesia is induced in American hospitals, though it has to be admitted that a great number of American operators are still deplorably lax about this matter.

Another step forward which will probably prove an important one is the organisation of a special National Society of Anæsthetists at the recent meeting of the American Medical Association. This organisation has been cordially welcomed in the professional press of the United States, as reforms in hospital practice are always sure of favourable comment.

